# Supplementary material for: Identification of photoperiod-induced specific miRNAs in the adrenal glands of Sunite sheep (Ovis aries)
Source: Front Vet Sci. 2022 Jul 22;9:888207. doi: 10.3389/fvets.2022.888207 (PMC9354845; doi:10.3389/fvets.2022.888207)
Supplement: Supplementary file 1 [file Data_Sheet_1.ZIP › Supplementary file/miRDeep2 file.zip!/miRDeep2 file/Know_Structure/LP42A2/oar-mir-200b.pdf]

miRBase precursor : oar-mir-200b
Total read count : 207
oar-miR-200b read count : 207
remaining reads : 0

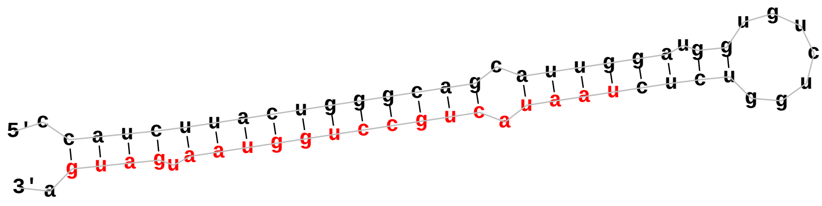

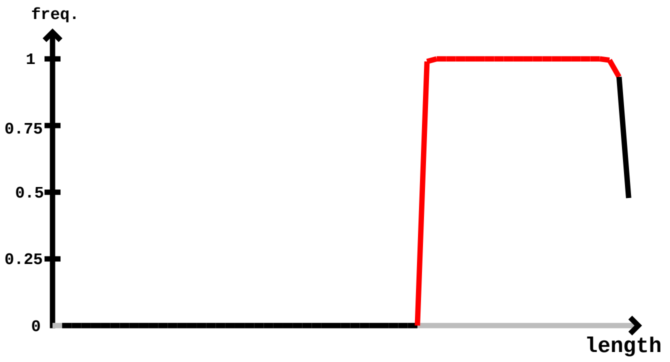

oar-miR-200b

```
5'- ccaucuuacugggcagcauuggauggugucuggucucuaauacugccugguaaugaugaa   -3'    exp
    .((((((((((((((.((((((.((.......)))))))).)))))))))))).)))).     reads      mm         sample
    ....................................uaauacugccugguaauga...     1          0          seq
    ....................................uaauacugccugguaaugau..     13         0          seq
    ....................................uaauacugccugguaaugaug.     94         0          seq
    ....................................uaauacugccugguaaugauga     97         0          seq
    .....................................aauacugccugguaaugauga     2          0          seq
```
